# *“We can hardly even do it nowadays. So, what's going to happen in 5 years from now, 10 years from now?”* The health and community care and support needs and preferences of older people living with HIV in Ontario, Canada: a qualitative study

**DOI:** 10.1002/jia2.25978

**Published:** 2022-09-29

**Authors:** Kate Murzin, Elizabeth Racz, D. M. Behrens, Tracey Conway, George Da Silva, Eimear Fitzpatrick, Joanne D. Lindsay, Sharon L. Walmsley, the PANACHE study team

**Affiliations:** ^1^ Realize Toronto Ontario Canada; ^2^ Toronto General Hospital Research Institute University Health Network Toronto Ontario Canada; ^3^ MAP Centre for Urban Health Solutions Unity Health Toronto Toronto Ontario Canada; ^4^ Department of Medicine University of Toronto Toronto Ontario Canada

**Keywords:** HIV care continuum, quality of life, social support, structural drivers, North America, community

## Abstract

**Introduction:**

The population of people living with HIV (PLWH) is ageing consequent to effective treatment and a steady stream of new diagnoses among older adults. PLWH experience a greater burden of age‐related comorbidities and poorer social determinants of health compared to their HIV‐negative peers, yet comprehensive requisites for care and support as PLWH age remain poorly understood. Preferences And Needs for Ageing Care among HIV‐positive Elderly people in Ontario, Canada (PANACHE ON), explored the health and community care and social support needs and preferences of a diverse group of older PLWH (age 60+) and described life course experiences among older PLWH that shape these needs and preferences and whether they are met.

**Methods:**

PANACHE ON was a qualitative community‐based participatory research study. In‐person focus groups using a semi‐structured interview guide were co‐facilitated by pairs of trained older PLWH from July to October 2019. Purposive sampling bolstered the inclusion of communities disproportionately affected by HIV in Ontario. Descriptive analysis was used to summarize demographic data; participatory data analysis was conducted by a subset of the research team, with transcripts double‐coded and analysed using NVIVO 12 Plus.

**Results:**

A total of 73 PLWH participated, 66% identified as men. The mean age was 64 years (range 55–77) and median time living with HIV was 23 years (range 2–37). The current and anticipated needs of older PLWH, many of which were only partially met, included necessities such as food and housing, mobility and sensory aids, in‐home support, social and emotional support, transportation and information. Three experiences—trauma, stigma and uncertainty—intersected in the lives of many of our participants, shaping their needs for care and support, and impacting the ease with which these needs were met.

**Conclusions:**

Unmet health and social needs and limited control over the availability and accessibility of ageing‐related care and support due to resource constraints or reduced capacity for self‐advocacy results in anxiety about the future among older PLWH, despite their well‐developed coping strategies and experience navigating systems of care. These study findings will inform the development of the first national needs assessment of older PLWH in Canada.

## 1 INTRODUCTION

The number of people living with HIV aged 50 or older (PLWH50+) worldwide was projected to reach 7.5 million in 2020 [1], and in sub‐Saharan Africa alone, will surpass 9 million by 2040 [2]. By 2030, it is estimated that one in four PLWH in high‐income settings will be at least 60 years old (PLWH60+) [3]. In 2015, the median age of PLWH in Ontario was 48 and an estimated 4300 PLWH were 55 years old or older [4]. Given the excess burden of age‐related comorbidities on PLWH [5], relevant ageing‐related care and supports are long overdue.

The 2006 ROAH study, the first to assess the comprehensive needs of ageing PLWH [6], found that multi‐morbidity was the norm, depression and stigma common, and PLWH50+ had more unmet practical and emotional support needs than HIV‐negative older adults. Since then, research in several jurisdictions has increased understanding of the lived experiences of older PLWH and their impact on ageing‐related needs for care and support [7, 8, 9, 10, 11, 12, 13].

A comprehensive needs assessment of older PLWH to inform policy and practice change has never been conducted in Canada. Four qualitative studies have provided most of the information available on the multi‐dimensional wellbeing of PLWH50+ in Ontario. Of these, only one garnered detailed narratives on service use and unmet needs, engaging 11 PLWH50+ in one city in 2009 [14]. Data from these participants were also used to explore their housing and mental health experiences [15, 16]. A second examined factors contributing to disability and uncertainty among PLWH50+ in Southern Ontario [17, 18]. The remaining two explored successful ageing among PLWH50+ residing in two urban settings producing a significant body of literature detailing resilience strategies, environmental barriers to ageing well and trajectories of episodic disability over time [19, 20, 21, 22, 23, 24, 25, 26, 27, 28, 29, 30, 31]. Cultural considerations [32] and specific psychosocial issues affecting older PLWH in Ontario (i.e. cognitive health [33, 34], peer support [35], stigma [36, 37] and housing and food security [38]) have been investigated in isolation.

A literature review reveals gaps in our understanding of the comprehensive care and support needs of older PLWH in Ontario. Prior studies engaged PLWH50+ so findings were shaped by the responses of predominantly middle‐aged participants, most of whom resided in urban settings in Southern Ontario. Involvement by women and racialized people has been underreported or lacking; data from the single study focused on service use are outdated; and an explicit focus on “ageing successfully” may have the limited analysis of the impact of poor social determinants of health among older PLWH lacking personal resilience strategies.

To address these gaps and inform development of the first national‐level comprehensive needs assessment survey of older PLWH in Canada, we conducted a qualitative pilot study. The purpose was to describe the health and community care and social support needs and preferences of a diverse group of PLWH60+ from across Ontario. Additionally, we aimed to describe the life course experiences which shape their needs and the extent to which they are being met.

## 2 METHODS

Preferences And Needs for Ageing Care among HIV‐positive Elderly people in Ontario, Canada (PANACHE ON) was a community‐based participatory research (CBPR) study. Approximately half of our 32‐member research team identified as older PLWH (*n* = 18), many with academic research training, extensive CBPR experience and/or front‐line roles in community‐based HIV organizations (CBHO). The team was rounded out by clinician–researchers (*n* = 2), CBHO staff (*n* = 8) and academic researchers (*n* = 4). Our work was guided by CBPR principles like equitable partnerships, community and researcher capacity‐building, mutual respect for all forms of knowledge and a commitment to translating research into action [39]. Most study activities, including data collection and primary analysis, occurred between May 2019 and August 2021.

PLWH60+ were recruited through CBHOs using posters and social media channels. Nine focus groups were planned; five were open to all PLWH60+, and four were designated for “priority populations”—communities disproportionately affected by HIV in Ontario [40] including those who self‐identified as: gay, bisexual and other men who have sex with men (gbMSM); women; racialized persons; and people with drug use experience. Focus groups enable participants to build on or challenge ideas raised by others and provide the added value of peer support, a need well‐documented among PLWH and older adults [41, 42, 43].

A semi‐structured interview guide was developed collaboratively with PANACHE's Research Tool Development working group; content was based on relevant themes emerging from HIV and ageing literature and a prior concept mapping exercise engaging the full PANACHE team [44]. Seven intersecting themes were explored: social supports and stigma; sexual health; physical health; mental health; housing; food security; and finances. Existing partnerships with CBHOs were leveraged to support recruitment and establish comfortable meeting spaces. Eight peer research associates (PRAs) were hired and trained in focus group facilitation and co‐facilitated in pairs.

Nine focus groups (5–13 participants) were conducted between July and October 2019. Self‐reported demographic data, including birth year, estimated years living with HIV, gender identity, sexual orientation, racial background, household composition, income source and housing type, were collected using an anonymous survey prior to each focus group.

Focus group data analysis was conducted by a sub‐committee of the PANACHE research team, including PLWH, and guided by DEPICT [45], a six‐step model that facilitates participation in qualitative data analysis by stakeholders with differing levels of research literacy and experience. The model prioritizes collaboration and transparency. Two members engaged in “dynamic reading” of the transcripts, an inductive process for identifying ideas emerging from the focus group discussions; ideas were organized under the seven themes outlined in the interview guide and defined and/or exemplified using transcript data. Through “engaged codebook development,” 74 ideas were consolidated into 25 codes by the analysis team. In the “participatory coding” phase, each transcript was coded independently by two team members. New codes were added as ideas emerged that did not fit elsewhere. Coded data were consolidated in NVIVO 12 Plus. “Inclusive review and summarizing of categories” as well as “collaborative analyzing” were done through 13 virtual analysis team meetings, as necessitated by COVID‐19‐related restrictions.

This study received ethics approval from the University of Toronto HIV REB Protocol #: 00037889. Participants provided written informed consent. PRAs facilitated confidentiality discussions at the start of each session.

## 3 RESULTS

The PANACHE ON focus groups included 73 participants whose demographics are displayed in Table 1. The mean age of participants was 64 years and the mean time since HIV diagnosis was 22 years. Two‐thirds of participants self‐identified as men and one‐third as women including one trans woman. Half identified their race as White. Most rented their dwelling or lived in supportive housing; and living alone was the norm (*n* = 45, 59%). Most relied on one or more public or private benefit programmes for income.

**Table 1:** Summary data PANACHE ON participants, *N* = 73

Characteristic	*n* (%)
Age, year	
Mean	64
Range	55–77
Gender identity^a^	
Man	49 (66%)
Woman	21 (28%)
Transwoman	1 (1%)
Two spirit	2 (3%)
Not specified	1 (1%)
Sexual orientation	
Gay	34 (47%)
Straight (heterosexual)	25 (34%)
Bisexual	8 (11%)
Asexual	3 (4%)
Not specified	3 (4%)
Racial background	
White	39 (53%)
Black	14 (19%)
Indigenous	6 (8%)
East Asian	3 (4%)
Latin‐American	3 (4%)
Southeast Asian	2 (3%)
Mixed race	2 (3%)
South Asian	1 (1%)
Not specified	3 (4%)
Length of time living with HIV, years	
<10	11 (15%)
10–19	16 (22%)
20–29	27 (37%)
30+	18 (25%)
Unknown	1 (1%)
Housing	
Rent	51 (70%)
Own	12 (16%)
Housing facility	3 (4%)
Room (in house)	2 (3%)
Not specified	5 (7%)
Household composition^a^	
Live alone	45 (59%)
Live with a spouse or partner	14 (18%)
Live with a child/children	8 (11%)
Live with extended family	3 (4%)
Live with unrelated people	5 (7%)
Not specified	1 (1%)
Employment	
Employed full time	2 (3%)
Employed part time	9 (12%)
Income source	
Employment only	2 (3%)
One benefit source^b^	32 (44%)
Part time employment + at least one benefit	9 (12%)
Multiple benefit sources	29 (40%)
Not specified	1 (1%)

^a^Values do not equal the total number of study participants (*n* = 73) as some participants selected more than one response.

^b^Benefit sources reported included: Ontario Works (OW); Ontario Disability Support Program (ODSP); Old Age Security (OAS); Guaranteed Income Supplement (GIS); Canada Pension Plan (CPP); Canada Pension Plan—Disability (CPP‐D); Private Pension; and Private Disability Pension.

### 3.1 Current needs of older PLWH in Ontario

Our focus group participants described varying needs ranging from basic survival to community inclusion. Affordable, safe, secure housing and healthy, plentiful food were the most cited needs, often arising because they were partially unmet.

Physical and mental health needs included medication, medical supplies, mobility aids, dental work, eyeglasses, hearing aids, psychological therapy and crisis response services. A few described needing another person physically present in their living space, or close by, in case of illness or injury, specifically falls. The need for more information on ageing (e.g. care options, financial planning and service navigation) was explicitly raised and a lack of clear understanding of government benefits was observed.

Several social and emotional needs were mentioned, including companionship, emotional, spiritual and peer support, and inclusion in the HIV community. Several participants linked their need for community engagement with the practical need to be mobile (e.g. to see friends and to get to their local CBHO), which was becoming more difficult with age.

### 3.2 Needs: met and unmet

Participants reported using health services, including pharmacy, primary care, specialist care, dentistry/denturism, vaccination, laboratory, occupational therapy, vision and hearing services, and cognitive assessment and support. Many described seeking or accessing mental healthcare, including crisis intervention, psychiatry and counselling. Massage was the most mentioned complementary therapy need.

Not all participants’ health needs were being met by all these services and barriers to fulsome service access included social determinants of health (e.g. inability to afford uninsured services, including complementary therapy, vision and hearing services, dental work and certain vaccinations), provider issues (e.g. concerns about confidentiality, lack of knowledge about HIV and ageing) and structural challenges (e.g. siloed care, subjective assessments of eligibility for services and lack of just‐in‐time services). Several people underlined the need for coordinated models of care and patient–provider relationships where they felt heard. One long‐term survivor felt abandoned by his local CBHO due to an unmet need for crisis care:

“when I've called, “oh well we can't today, maybe we can bring someone out to you tomorrow”, or…“why should we provide you that service?” So, I lay there no food in the house, medically sick, and unless I call the ambulance, which I don't have the money to pay, I've got two bills right now… I'm locked down.” (Group 1, Participant 7)

Workarounds were often used to partially address unmet health needs, for example accessing free rehabilitation and complementary health services through local CBHOs, although these services were available on a time‐limited basis or too infrequently. One modest income pensioner used a monthly plan to pay a substantial hearing aids co‐pay. Compensatory strategies, such as recording passwords and employing physical or electronic reminders for appointments and medication, were used by several to manage lapses in memory.

Subsidized housing was relied upon by a large proportion, and although these services alleviated the pressure of paying market rent and prevented homelessness, they were the source of significant challenges. One woman described waiting 16 years for a subsidized unit and then being told it might take another 3–5 years for seniors’ housing placement. Several described community housing living environments as extremely stressful due to the consistent presence of law enforcement and emergency services, crime, infestations, disrepair and bully managers.

Food banks and community meals, often provided by CBHOs, were used by many to address basic nourishment needs. Even so, participants lamented the lack of food choices and the need to ration the limited quantity of food provided. CBHOs were also a significant source of needed social and peer support among most participants. Even so, ageism within the HIV community and gaps in relevant HIV services (e.g. person‐to‐person appointment reminders and groups for PLWH60+) were raised.

Participants’ unmet needs differed with their community size. Those residing in a major urban centre referred to “city stress” and feeling more vulnerable to victimization with age. gbMSM in one small city grieved the loss of local gay bars limiting spaces for socializing. Many living outside cities loved their surroundings but access to community services was limited by transportation issues and capacity within small CBHOs.

### 3.3 Anticipated future care and support needs and preferences

There was a strong preference for ageing in the community, but many questioned the suitability of their current housing in the future. Several participants spoke of the potential need to move with age—whether to liquidate assets for retirement, downsize in response to increasing financial pressures or find housing to accommodate declining functional status (e.g. to be closer to transit services if they can no longer drive; to eliminate property maintenance)—but questioned available housing options.

Many older PLWH were uncertain as to their ability to live alone due to expected changes in mobility or capacity to perform instrumental activities of daily living (iADLs) (e.g. home maintenance and cooking), especially those living rurally. One respondent pre‐emptively moved into a building with iADL supports in anticipation of future needs. A few mentioned family supports may enable ageing in place more easily, while others lacked informal caregiver support. Concerns were raised around the need to hide their sexual orientation or HIV status if relocating to a seniors’ complex, subsidized housing building or long‐term care centre for fear of stigma from other residents and/or staff. Participants questioned the safety of residential care for seniors, regardless of HIV status, due to neglect, understaffing, victimization and lack of regulatory oversight.

Several also worried about increasing isolation and loneliness with age or feared dying alone. Impaired vision and hearing, mobility challenges and declining function were potential contributing factors. At least one participant reported missing local CBHO social events due to episodic illness, and another worried worsening mobility would increase their reliance on visits from others.

### 3.4 Lived experiences shape ageing care and support needs and preferences

Three intersecting themes emerged in our analysis—trauma, stigma and uncertainty—and their cumulative burden affected participants’ present‐day and future care and support needs, and the likelihood these are, or will be, met. Traumatic experiences across the life course often resulted in the loss of financial security and social and caregiving support. Experiences of HIV stigma and ageism increased isolation and reliance on formal services, and amplified concerns about discrimination in care settings. Current and common anxiety about meeting basic needs led to concerns about future resources.

#### 3.4.1 Past trauma and loss have compromised the safety net

Our participants described life‐long traumatic experiences, often related to HIV, including diagnosis with poor prognosis, abandonment by loved ones after disclosure and significant losses (i.e. people, aspirations, sense of security and capital) due to illness; these experiences had both an immediate and enduring impact. Loss of intimate relationships decreased social capital, and for some, prohibited the development of new connections. Many worried about inadequate social support for ageing at home in the absence of practical support from their family of origin. One gay man living long‐term with HIV described the challenge of seeking support from those with whom you lack an established history:

“I have been HIV for a very extended period of time, and have lost a lot of my connections, people have died. Period, that is our reality. As an individual, you don't have that sort of formative relationship with new people. So the dynamics in terms of looking for support from them is a bit different, it almost is nonexistent.” (Group 1, Participant 4)

Hopelessness, bad advice and/or episodic illness led to material losses (e.g. earning potential and assets) for some, compromising their financial security. Many described living in poverty having given up opportunities to earn and save, dispensing with assets and being forced into an inescapable reliance on disability benefit programmes. Uncertainty about meeting basic needs for food and shelter was a daily reality for many. A gay man who was “new to HIV” described the swift and significant financial impact of starting disability benefits:

“But I had RRSPs and everything, I saved all that, but… and I had a legal practice. But I got really sick when it happened. It wiped everything out.” (Group 7, Participant 4)

Past HIV‐related trauma impacted mental health and permanently compromised the social and financial safety nets of participants in our study shaping their emotional and practical care and support needs in older adulthood. Community‐based services, especially CBHOs, helped meet social, informational and emotional needs, especially for those with limited informal support. Community support programmes for seniors helped with loneliness even for those with supportive partners:

“There's a woman that works… a very young lady, a 22‐year‐old, she's wonderful. And she now calls me every day … Monday to Friday, at 4:30 and chats with me. Do you know what a ray of sunshine that has made in my life.” (Group 3, Participant 7, Gay Man)

Programmes designed to address basic needs directly (e.g. subsidized housing and food banks) were insufficient, forcing the individual to make ends meet. Personal strategies to partially meet survival needs with limited resources included: living rurally, rationing food, using public utilities (e.g. internet in libraries) and paying with credit cards and high‐interest loans; but these trade‐offs added to participants’ burden.

#### 3.4.2 Experiences of stigma shape assumptions about ageing care and support

Discriminatory treatment from others was a common experience. HIV stigma was enacted in one‐on‐one relationships and communities.

Participants also described the impact of embodying multiple intersecting stigmatized identities based on race, class, HIV, age, sexual orientation and drug use, such that they were unable to determine which biases were responsible for impeding access to resources or support. A racialized gay man described multiple layers of stigma in his cultural community, saying:

“I'm from Jamaica myself, and trust me, okay, it's homophobic, it's HIV‐phobic, it's every phobia you can conceive…” (Group 8, Participant 3, Racialized Gay Man)

Few participants described experiencing or observing HIV stigma when accessing health services; however, several shared the belief that doctors were dismissive towards people ageing with HIV, with one woman saying:

“You know, 60‐some years old and you've got HIV, so we're not going to bother looking into that… but it seems to be happening at a younger age than [*sic*] people that are positive because they're going well you're on bonus time anyway and…” (Group 5, Participant 1)

Several participants, especially gbMSM, adamantly refused to internalize HIV stigma and ended relationships to protect against it. In contrast, internalized stigma was evident in the narratives of others who described feeling ashamed about living or dying with HIV/AIDS or were hard on themselves for not disclosing to loved ones. One woman living with HIV long‐term described how learning that Undetectable = Untransmittable changed her whole view of herself:

“I phoned my partner right away and I said “I'm clean.” And he goes “what are you talking about?” I said “I can't give it to you. I'm clean now.” And for 20‐some years I felt dirty because that's how the public perceived me, that because I had AIDS at that one time, that I was dirty.” (Group 3, Participant 2, Woman)

Ageist assumptions and behaviours transcended multiple interactions and environments from dating to healthcare and community services to society. Participants described instances where they were tokenized, rejected and ignored by individuals and institutions. One individual observed that ageism in the HIV community is not addressed with the same urgency as other forms of stigma:

“… and it's really strikingly so around the issue of age too, in this age… in this era of identity politics, and ageing issues just are not there, they're not ever there and you usually get a blank look when you bring them up at the conferences and so on.” (Group 4, Participant 5, Diagnosed < 10 years)

Internalized ageism and ableism were also observed. One participant depicted ageing as the process of “transitioning from a productive life… to one where…you become someone who requires care.” Another did not discuss ageing or their chronic illness with family to avoid being a “hindrance.” Increasing difficulty with activities of daily living was “embarrassing.”

Both internalized stigma and past and present experiences of discrimination increased anticipated stigma. We observed widespread fear of neglect and mistreatment in ageing care that may impact willingness to engage regardless of need.

#### 3.4.3 Uncertainty makes planning for care and support more difficult

Some participants described experiencing uncertainty in their daily lives. Constant worry about paying for food and medicine tested their coping ability. One individual reflected on the need to adapt to the onslaught of new age‐related illnesses:

“…it just keeps evolving and it takes time to accept each one of these conditions. And we seem to hold in that pattern of waiting to be able to accept it and then something else happens.” (Group 1, Participant 7, Gay man living long‐term with HIV)

Overall, participants did not see their circumstances improving with time. Ageing was seen as an anxiety‐provoking process of becoming more vulnerable and having insufficient resources to compensate for decreasing personal capacity. Participants used the words dread, worry, concern and panic to describe anticipated ageing‐related changes in financial security, health, safety, autonomy, independence, care and social support.

Uncertainty about the availability and accessibility of appropriate formal ageing care and supports was common. In the women's focus group (Group 6), the eligibility criteria for assistive devices subsidies were not perceived as transparent or equitable. Some participants were unaware of existing subsidized or low‐cost formal home and community care services that support ageing in place. Many worried their current income or future pension would be insufficient to meet basic needs or permit access to reputable residential ageing care. Inability to exercise control over how their current or future needs were met contributed to uncertainty and anxiety. One woman described giving up meaningful occupations while staying with family after a series of falls:

“I don't want to give up my independence. Like since moving in with my brother I've given up my artwork, it's sitting at home not being done, I've given up my writing, I don't have a computer to work on, you know, I'm sitting there… I'm isolated with him for my safety, but I'm not able to do any of the things that make my life worthwhile.” (Group 2, Participant 4, Heterosexual Woman)

## 4 DISCUSSION

This qualitative study aimed to identify the care and social support needs and preferences of a diverse group of PLWH60+ in Ontario, Canada; and to describe lived experiences shaping these needs and affecting whether they are met. Our findings add to the existing body of literature since we purposively engaged PLWH60+, rather than PLWHIV50+; as well as those residing outside urban centres and those identifying as women and/or racialized persons.

We found that the basic needs of older PLWH, including safe, affordable housing and high‐quality sufficient food, are only partially met by the existing social service infrastructure; previous studies have reported similar findings [9, 10, 11, 14, 38]. Government subsidies for vision and hearing aids are inadequate resulting in older PLWH having to pay out‐of‐pocket or go without. Older PLWH also rely on improvised self‐management strategies to deal with memory changes since services in this domain are largely non‐existent, especially outside urban centres [33]. Social and practical needs are met informally by family and friends in some cases, and predominantly by CBHOs among those with no or small social networks, but there is a lack of tailored programming for older PLWH [17, 28, 29]. The needs of older PLWH documented over the last decade continue to go unmet and are now much more pressing due to the growing proportion of PLWH60+.

The needs and preferences of our older PLWH are shaped by past and present experiences of trauma, stigma and uncertainty, a life course triad also described by other researchers [14, 15, 17, 19, 20, 31]. As the majority were diagnosed with HIV in their prime working and relationship‐building years and currently rely primarily on government income supports, the adequacy of financial and social capital to live in comfort and dignity is a significant source of uncertainty [17, 18, 31]. Despite a strong preference for ageing in place, there is much unknown about how this will be operationalized.

The care and support needs raised in this study reflect those identified by community‐dwelling older people with other chronic conditions or multimorbidity [46, 47]. Both experience uncertainty about the future, sensory impairment, difficulties with iADLs, social isolation and loneliness, and challenges navigating uncoordinated services; in response, they employ self‐management and coping strategies, turn to informal caregivers and peers for support, and seek technological aids for mobility, vision and personal safety [46].

Older PLWH require care and support that accounts for a different life course trajectory than the ageing general population consequent to the impact of trauma, intersecting forms of stigma and uncertainty on their mental health [15, 36, 48, 49, 50]. The practical realities resulting from this triad of lived experiences, including their potential to shape the needs of older PLWH and affect whether these needs are fully met, have received less attention. A significant source of uncertainty is how needs will be met if participants become unable to live independently, as previously documented [18]. Many reject long‐term care or seniors’ housing for fear of HIV stigma and/or homophobia [9, 51, 52]. Although we did not hear many examples, the literature documents experiences of discrimination in healthcare and other institutional settings as common among older PLWH [14, 53]. Furthermore, Koehn et al. describe that, without careful planning, communal living environments can trigger stress among PLWH with trauma histories [54]. Based on their past experiences, residential seniors’ care is not viewed as being safe by older PLWH.

The alternative is to access support to age in place, at home, but this option is also fraught with barriers. As in previous studies involving PLWH and sexual minority older adults [18, 55, 56], some participants worried about having inadequate social capital to address practical support needs. Ploeg and colleagues found that HIV‐negative older people with multimorbidity were “heavily dependent on their family caregivers for many kinds of support, without which, they would be unable to continue living in the community” even though the majority of those interviewed had an income greater than $40,000/year [47]. Older PLWH in our study with multimorbidity lacked both practical support from social networks and the financial means to pay for non‐insured formal care.

Our findings are consistent with needs assessments conducted with older PLWH in other jurisdictions. Income, housing and food insecurity were common in older PLWH in other high‐income countries [9, 10, 11, 12, 53, 57, 58]. Financial issues among older PLWH in low‐ and middle‐income countries affected access to HIV care in Uganda and quality of life in Brazil [59, 60]. Many ROAH 2.0 respondents indicated some difficulty with activities such as housework, getting to places outside walking distance and shopping [9, 10, 11]. Many of the current and anticipated support needs we identified, including access to appropriate mental health services, practical support within the home and opportunities to socialize, were also highlighted [9, 11, 14, 15, 17, 29]. The need for publicly funded sensory and dental care services for pensioners in Canada was discussed frequently among participants; these services were also identified as important components of a comprehensive care programme for older PLWH in the United States [8].

Our research demonstrates that older PLWH draw on their internal resources—knowledge, skills, strategies and self‐efficacy—developed over years of living with a chronic illness, to plan for and manage ageing‐related changes. As in previous studies, our participants described an array of practical and emotional coping strategies [15, 19, 20, 21, 22, 24, 28, 29]. Despite their tenacity, upstream approaches are needed to ensure that all older PLWH have their needs met and can age with the same respect, honour and minimum of stress as their non‐HIV‐positive peers.

In addition to our primary findings, we noted a few population‐specific differences. First, many of the older gbMSM living with HIV were outspoken about their HIV status as a strategy to identify and dismiss people who might stigmatize them, a strategy previously coined as pruning social networks [20]. Similarly, Kia et al. found that older gbMSM living with HIV used purposeful disclosure of their sexual orientation and HIV status to push back against stigmatizing service provider behaviour [61]. These findings suggest a preference for models of care that centre the experience of living with HIV among older gbMSM. Second, older PLWH living outside urban centres faced different challenges; they relied on infrequent transit services to access support programming and had greater concerns regarding HIV stigma, barriers also cited by rural‐dwelling older PLWH in the United States [62]. These and other differences between population groups of older PLWH warrant more study.

This qualitative project was an important precursor to a planned national needs assessment of PLWH60+ in Canada. Inherent limitations in this pilot, such as the use of anonymous surveys to collect participant demographic information, limited our ability to contextualize individual participants’ responses, except in cases where the speaker's own narrative identified aspects of their identity.

Despite our targeted recruitment strategy, our findings are based on the narratives of a small sample and not generalizable to all PLWH60+ in Ontario, especially those who are transgender, rural dwelling, not connected to community organizations or newly diagnosed. Furthermore, as two‐thirds of our participants were HIV long‐term survivors, our findings likely confound care and support needs related to ageing with HIV with those of long‐term survivorship [63]. Ageing with HIV may be significantly different for people diagnosed in the era of improved HIV treatment.

Our semi‐structured interview guide did not prompt study participants to frame their needs for care and support in the context of formal health or social support services. Barriers to service access arose organically in the discussion, baring the question which explored participants’ ability to pay for services, support and resources to help meet their self‐identified needs. As physician‐ and hospital‐based services are paid by Ontario's provincial health insurance programme, the focus was more on needs related to the social determinants of health.

We illustrated the multidimensional needs of older PLWH in Ontario and described how a combination of life experiences common to PLWH60+ contributes to discrepancies in access to care and support services that address these needs in older adulthood. These findings draw attention to some of the existing policy and practice gaps and barriers for older PLWH in Ontario. Income support programmes, both disability‐ and ageing‐related, and government‐funded health insurance, for example, do not provide adequate benefits coverage to meet the holistic health needs of low‐income older people, including many PLWH. CBHOs are an important and safe source of emotional and peer support for many older PLWH with small social networks, but lack programming specifically designed for older PLWH. Our study findings will inform advocacy in these areas and justify our next step to quantify the prevalence of certain experiences and the frequency of unmet service needs.

## 5 CONCLUSIONS

PLWH experience ageing with the addition of three major burdens: trauma, stigma and uncertainty, which impact their health and social care and support needs and may not intersect as commonly among their ageing HIV‐negative peers. Despite the resilience demonstrated by PLWH60+ in this study, systemic and structural barriers continue to contribute to inequities in the potential to meet ageing‐related needs enabling PLWH to age in comfort and dignity. Changes to health and social policy are required to address these gaps.

## COMPETING INTERESTS

The authors declare no competing interests.

## AUTHORS’ CONTRIBUTIONS

KM and SLW are Co‐Principal Investigators of PANACHE and coordinate all activities of the research team. KM and ER trained PANACHE ON PRAs, implemented the study protocol, oversaw the participatory data analysis and interpretation process and prepared the draft manuscript. SLW provided research mentorship to KM and ER, participated in data analysis and interpretation, and edited the manuscript. ER managed study data in NVivo. DMB, TC, GDS, EF and JDL participated actively in data analysis and interpretation and provided feedback on the manuscript.

## FUNDING

Ontario HIV Treatment Network—CBR‐1106.

## Data Availability

The data that support the findings of this study are available on request from the corresponding author. The data are not publicly available due to privacy or ethical restrictions.
